# High-Resolution Remotely Sensed Small Target Detection by Imitating Fly Visual Perception Mechanism

**DOI:** 10.1155/2012/789429

**Published:** 2012-01-15

**Authors:** Fengchen Huang, Lizhong Xu, Min Li, Min Tang

**Affiliations:** College of Computer and Information Engineering, Hohai University, Nanjing 210098, China

## Abstract

The difficulty and limitation of small target detection methods for high-resolution remote sensing data have been a recent research hot spot. Inspired by the information capture and processing theory of fly visual system, this paper endeavors to construct a characterized model of information perception and make use of the advantages of fast and accurate small target detection under complex varied nature environment. The proposed model forms a theoretical basis of small target detection for high-resolution remote sensing data. After the comparison of prevailing simulation mechanism behind fly visual systems, we propose a fly-imitated visual system method of information processing for high-resolution remote sensing data. A small target detector and corresponding detection algorithm are designed by simulating the mechanism of information acquisition, compression, and fusion of fly visual system and the function of pool cell and the character of nonlinear self-adaption. Experiments verify the feasibility and rationality of the proposed small target detection model and fly-imitated visual perception method.

## 1. Introduction

With the fast development of sensor technique, the high spatial/spectral resolution remote sensing systems are more and more deployed. For example, the spatial resolution of American space remote sensing platform QuickBird, Worldview has achieved meter level or submeter level of resolution. The spatial resolution of military reconnaissance satellite KH-12 and 8X-1 has achieved a resolution of 0.1 meters level. The American space AVIRIS spectrum resolution amounts to 10 nanometers while the Trwis-3 spectrum resolution is even higher, amounting to 6 nanometers, and the band number surpasses 380. The China's 12th five-year plan endeavors to push the development and implementation of the observation project of the high resolution to the ground level (implemented in the 11th five-year plan) and concentrates on the emerging research focus on the basic theories and key techniques of the high-resolution remote sensing target, space environment feature analysis, and high reliable automatic interpretation to meet the requirement of national security and massive demand of the socioeconomic development.

Differing from the observation for the larger-scale and high-resolution remote sensing at ground level, the present small target detection technology has faced huge challenges due to increasing types of recognizable ground object, high density, complex target detection environment caused by synonyms spectrum of same object (or the same spectrum of different object) and scarce target prior information.

Remote sensed small targets in high resolution occupy little pixel areas while they contain rich detail texture features (such as airplane, suspicious items, and military installations). In contrast the precise and abnormal detection of hyperspectral remotely sensed materialized parameters barely has texture and shape structure information. Many researchers and institutes have made extensive efforts on this topic. The methods can be divided into three types: subspace-based methods, machine learning-based methods, and object-oriented methods.

The subspace transformation-based target detection method is a mathematical method based on image spectrum analysis. Hyvärinen and Oja proposed independent component analysis method in 2000 [1], and Chang [2] proposed target detection algorithm based on orthogonal transformation in 2005, both of which are based on subspace transformation. However, because this kind of linear space transformation method is not good for the abstraction of the high-dimensional feature information in image background, the sub-space method based on Kernel function is proposed. For example, Capobianco et al. proposed orthogonal sub-space mapping method based on Kernel function [3] and principal component analysis based on Kernel function, and so forth in 2009. Li and Yu adopted low-dimensional hyperplane structure to realize hyperspectral remote sensing image of anomaly target detection with better abstraction effect being achieved [4]. However because this type of method is confined with the problems of the selection of the Kernel function and the parameters estimation, it has a certain limitation.

Based on the machine learning method, the nonlinear no parameters' estimation of the background is achieved by abstracting background spectral high-dimensional statistical features according to the limited samples learning. Bruzzone and Carlin [5] in 2006 and Camps-Valls et al. [6] in 2010 proposed high-resolution remote sensing image target extraction method based on support vector machine, respectively. In 2009, aimed to the complex changeable feature of hyperspectral remote sensing image background, Mei et al. [7] proposed target detection method based on adaptive support vector machine. At present, this kind of method needs to be researched further because of the limitation of the selection of the sample data and the amount of the training samples.

Object-oriented target recognition and detection method is a kind of feature-level target detection method, which is paid much attention for expressing and applying semantic information features. In 2001, Blaschke and Strobl [8] first proposed object-oriented remote sensing ground object classification method, which is used by small target detection of high-resolution remote sensing image. In 2009, Sirmacek and Unsalan [9] proposed the method combining scale-invariant feature transform with graph theory. After that, Sirmacek and Unsalan [10] further put forward object-oriented detection method for building target in IKONOS image, but it has a certain difficulty in abstracting effective feature and combining reasonably to achieve the exact description of the targets for high-resolution remote sensing image of complex texture feature and rich details when it lacks prior information. Besides, Di et al. come up with the target detection method based on fuzzy integral [11].

In summary, the aforementioned methods have some limitations, and even they are in the face of great difficulties. In addition, vast amounts of image data also increase the computational cost of detection algorithm, which is not conducive to real-time requirements.

In recent years, our research group has been working on the bionic compound eye information processing and visual detection. In 2008 [12], for multiremote sensing platform monitoring, bionic compound eye information fusion system model and computational method was proposed. In 2009 [13], inspired by the fly compound eye the sequence image superresolution reconstruction and integration method is proposed. In 2010 [14], inspired by the fly the small-target detector algorithm in complex background is put forward.

The fly compound eyes composed of many small closely spaced eyes in the nature are taken into account. Flies can rely on the visual system with low-resolution and very limited computing power to accurately detect and track targets while flying in complex natural scenes with high speed. However this is still a big challenge for human who have mastered high-resolution imaging technology and the high-end data processing technology.

In order to provide a theoretical basis for designing the “low-order” target detector to percept the complex, “high-order” remote sensing image, this paper builds up a fly-imitated visual perception information expression model inspired by the fly visual system information acquisition and processing mechanisms, which is completely different from previous research strategy from a new perspective. The advantages of fly visual system, small target detection, and identification of high-resolution optical remote sensing make it unnecessary of precise modeling and priori information binding.

Aiming to solve the limitations of high-resolution remotely sensed small target detection methods, fly imitated visual information processing system pattern of high-resolution remotely sensed small target detection is built through the engineering simulation of perceptual mechanism of the fly visual system. Based on this pattern, fly visual information acquisition, compression, and integration mechanisms are simulated and the information expression of the fly-imitated visual perception based on “cartridges” is analyzed. Moreover, the fly visual “pool cell” function and nonlinear self-adaptations of neurons arrays are simulated, and then the small target detector and fly-imitated visual perception algorithm are proposed and designed.

At present, it is lack of the comparative study about fly visual neurons arrays, biological mechanism of pool cell, and engineering simulation of fly internal mechanism. The theory behind small target detector and algorithm design is also limited. Therefore, it is necessary to carry out the work on fly visual perceptual information expressing modeling by engineering simulation and the internal mechanism of the pool cell to support target detector design and fly visual neurons arrays and improve detection algorithm eventually.

## 2. The Basic Structure of Fly Visual System

**Figure 1:**
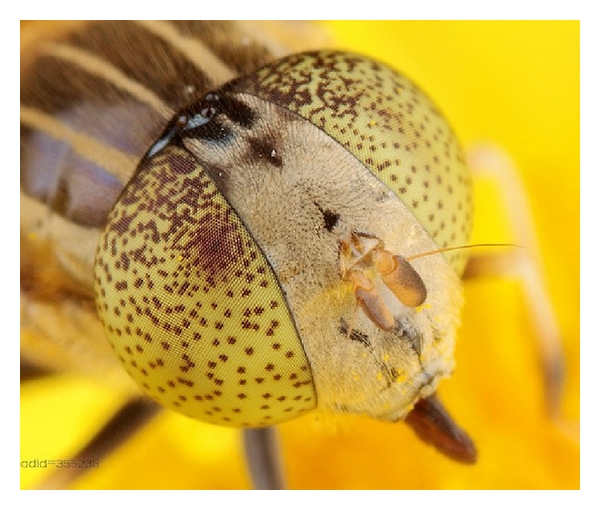
Fly compound eye.

**Figure 2:**
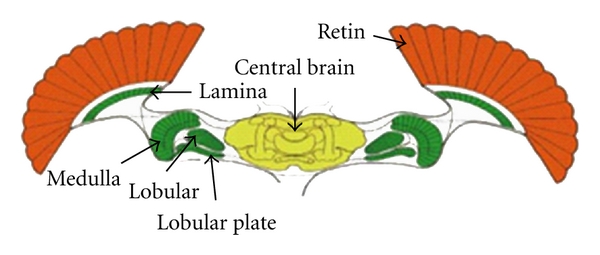
Section of fly visual channel.

Each compound eyes of flies are composed of about 3000–3200 small eyes, and each small eye is self-contained formed by the imaging system with cornea and crystalline dimension, light-sensitive retinal visual cells, and the optic nerve leading to the brain. Therefore each individual small eye can see things. The compound eye of fly is shown in Figure 1. The neural pathway sections from the primary visual information to advanced brain information processed from fly visual nervous system are shown in Figure 2.

Retina, lamina, medulla, lobular, lobular plate, central brain, and other tissues are mainly distributed in the neural pathway. The key of the fly visual system to achieve target detection is that the neural pathways formed by these organizations have highly nonlinear filtering properties.

The photoreceptor cells mainly complete information acquisition of the entire visual nervous system before the lamina and retina form the primary visual system processing stage of the compound eye. The cartridge structure compresses and fuses the obtained information and then spreads the information to the next high-order neural unit for processing by the LMC cells, which is composed by photoreceptor cells wrapped together with the LMC cell on lamina. LPTC, LGMD, STMD, and other high-order neuronal cell populations and pool cell populations are distributed in lobular, lobular plate, and brain. These nerve cells through the pool cell scheduling achieve the target perception and detection by background inhibition and target enhancement.

## 3. Fly-Imitated Visual Detection and Information Processing System Pattern

**Figure 3:**
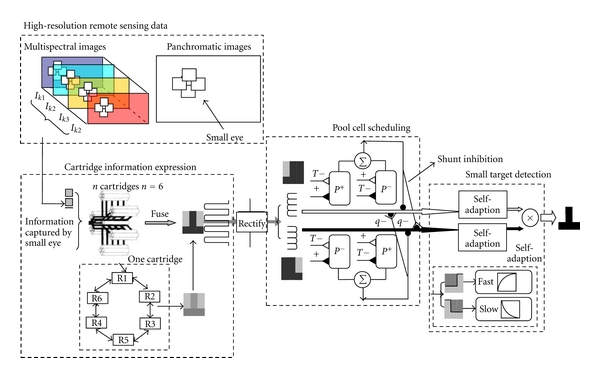
Fly-imitated visual detection and information processing system.

The fly-imitated visual detection and information processing system are shown in Figure 3. This system is built through comparative analysis among the fly visual system structure, mechanism, and the engineering simulation of internal mechanism in fly system. In addition, the system uses the following as the reference: the information processing mechanism of the visual neural pathways from the compound eyes retina imaging to brain center determining information, engineering simulated fly visual system mechanisms of acquisition, representation, and processing for natural scenes information. The system model mainly includes three parts: cartridge information expression, pool cell shunt and inhibition, and small-target detector [15].

The “cartridge information representation” section of fly visual system is different from the traditional “dynamic equation” analysis and accurate modeling. The so-called fly-imitated perceptual information expression models attempt to simulate the cartridge system for modeling, combining with background characteristics, targets and environmental characteristics, and performance requirements of target detection. The limited resolution of flies' compound eyes and the very limited computing resources are used for reference, which provide a theoretical basis for designing the “low-order” target detector to perceive complex, “high-order” remote sensing images. By simulating biological cartridge mechanism, thousands of small eye information can be fused, the information processing and computing transition from compound eye optical imaging to high-order neural array can be achieved, and the full-color data of high-resolution remote sensing image and multispectral data can be fused.

“Cartridge” information processing procedure mainly includes the following. (1) Information fusion: the total potential *V* of the cartridge is exponential adjusted by the difference between the membrane potential *v*_*k*·*l*_ of the single photoreceptor cell and the average membrane potential *v*_mean_ of the all photoreceptor cells in the cartridge. (2) Information compression: the information transmission way of ion diffusion is produced by the concentration difference of charged ions between cartridge structure and second-order neural LMC cells, and the information compression and feature extraction are achieved through the offset and confrontation of the luminance information within the local area.

“Pool cell scheduling” which is based on the characteristic information input achieves adaptive enhancement and inhibition effects of the feature by shunt inhibition to the two unipolar pool cells and one pair of bipolar pool cells in two sides of the compound eyes. The engineering simulation of the pool cell mechanisms is implemented by two steps. (1) Shunt inhibition: for different contrast polarity features channels, the fusion results of the features within the local area are used as inhibit components in the shunt inhibition procedure, which is good for the inhibition of the background texture feature. (2) Feature extraction: for the visible spectrum of the static image, the remote sensing image data coding method with comprehensive airspace and “map-in-one” spectral information is built to extract the characteristic information of remote sensing images.

“Small target detector” achieves target detection based on the biological mechanism of LPTC, LGMD, STMD, and other fly visual system target detection which refers to non-linear adaptive filtering process of high-order neural arrays, the correlation of opposite polarity, and so on. It mainly includes the following. (1) Nonlinear adaptive filtering mechanism: it simulates the biological mechanism of fast polarization and slow depolarization of high-order neural arrays, which can enhance the signals with low frequent and large magnitude of changes, and adaptively suppresses the texture signals with high frequent and small magnitude of changes. (2) Correlation of opposite polarity mechanisms: the correlation computation the edge features with opposite polarity in different spatial locations is used to obtain the effect of nonlinear adaptive target extraction without the high-accuracy description of target feature.

## 4. Fly Imitated Visual Perception Calculation of Small Target Detection

### 4.1. The Design of Virtual Compound Eye

The selection and design of virtual small eye should be set according to the requirements of fields applications. The satellite that equipped imaging equipment is regarded as a compound eye imaging system, while the panchromatic images and the multispectral images obtained can be mapped to “small eye” images in a logical concept.

For example, QuickBird is used as the research object to design the virtual small eyes: panchromatic and multispectral remote sensors equipped on QuickBird satellite use pushbroom imaging method, which can get one panchromatic band and four multispectral bands. The spatial resolution of panchromatic band is 0.61 meters and for multispectral band it is 2.44 meters while its spectral range is 450 nm to 900 nm. The local window containing only a small number of pixels is selected as single “small eye”. Five “small eyes” like the aforementioned ones are overlapping bundled, one of which is the center and the other four are overlapping with central “small eye” to form a sliding “small eyes group”. Multispectral bands use the sliding local window to extract the “small eye” images similar to panchromatic image.

Virtual “compound eye” system is divided into two types, one is composed of “small eye image” panchromatic image data, and another is composed of “small eye image” multi-spectral image data. In a certain phase, the virtual QuickBird compound eye system is composed of four groups of visible light “small eyes group” in low spatial resolution and one group of panchromatic “small eye group” in high spatial resolution.

### 4.2. Image Data Preprocessing

Because of the differences between panchromatic images and multi-spectral images in the spatial resolution, in fly-imitated compound eye system, remote sensing data always needs preprocessing, which requires different images registering each other; namely, it requires the image locations of the same ground object in different images overlapping. Many studies have proven that the phase-coherent model successfully explains the effectiveness of phase information sensed by biological vision and the stability for noise, brightness, and contrast changes. Therefore, the design is registered by phase coherence, which includes two steps: the first step is to obtain local energy function and each harmonic amplitude of spatio-temporal data by Gabor wavelet; the second step is to extract feature points to find the corresponding relationship between reference image and the registered image [16, 17].

### 4.3. The Biological Mechanism and Engineering Simulation of Small Target-Detected Neuron

In 1985, Egelhaaf [18] found that on the lobular plate of the fly visual system, there is a high-order neuron, small target motion detection (STMD), and pointed that the neuron has highly nonlinear filtering characteristics and high sensitivity for the mutant signals. In 2008, Wiederman et al. [19] built a small target detected neuron model according to the results of previous studies. The nonlinear adaptive mechanism, the central lateral inhibition mechanism, and the correlation of opposite polarity mechanism in the model can enhance the target feature while they can inhibit the background texture.

#### 4.3.1. Small Target-Detected Neuron Algorithm

**(1) Nonlinear Adaptive Mechanism**

**Figure 4:**
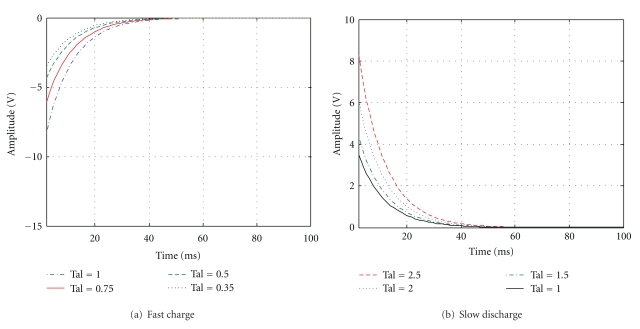
Nonlinear self-adaptive response characteristic circle.

Nonlinear adaptive mechanism enhances the mutant signals with low frequency and large amplitude of changes, inhibiting texture information with high frequency and low amplitude of changes. Taking the horizontal direction, for example (similarly, the vertical direction), for the output signal on_*h*_(*i*, *j*), the discrete form of the adaptive mechanism is represented as

(1) $$\begin{array}{cc} \text{if}\;\text{o}{\text{n}}_{h}\left(i,j\right) & <\text{o}{\text{n}}_{h}\left(m,n\right),\\ \text{o}{\text{n}}_{\mathrm{on}}^{\prime }\left(i,j\right) & =\text{o}{\text{n}}_{h}\left(i,j\right)-\exp\left(-\frac{\Delta s}{\varsigma_{1}}\right),\\ \text{if}\;\text{o}{\text{n}}_{h}\left(i,j\right) & \geq \text{o}{\text{n}}_{h}\left(m,n\right),\\ \text{o}{\text{n}}_{h}^{\prime }\left(i,j\right) & =\text{o}{\text{n}}_{h}\left(i,j\right)+\exp\left(\frac{\Delta s}{\varsigma_{2}}\right), \end{array}$$

where on_*h*_(*m*, *n*) is the characteristic response intensity for the (*i*, *j*) pixel in the channel on, and Δ*s* is the Euclidean distance between the two pixels which represents the interaction between the two pixels related to the distance. *ξ*_1_ and *ξ*_2_ are the response attenuation (enhancement) factor. Formula (1) shows that the signal intensity in position (*i*, *j*) is lower than that in the field around the signal, and the response will decay at the speed of exp⁡(Δ*s*/*ς*_1_); otherwise, it will increase at the speed of exp⁡(Δ*s*/*ς*_2_), namely, the rapid depolarization and slow repolarization in biological neurons, and the engineering simulated results are shown in Figure 4.

**(2) Central Lateral Inhibition Mechanism**

Central lateral inhibition mechanism can enhance the contrast between the signals. After the non-linear adaptive processing, the characteristic information of background is suppressed, and the feature information of target is retained. At this time, the central lateral inhibition mechanism can be used to enhance the retained target features, so the false alarm rate of the test results is reduced. Taking the channel on for example, in the local area *N*(*i*, *j*) with the field radius *r* (taking into account that the small target size in the horizontal or vertical direction is limited to 1 or 2 pixels, the radius of the local area is 1 ≤ *r* ≤ 2), the output of the channel on in the pixel position (*i*, *j*) is

(2) $$\text{o}{\text{n}}_{h}^{\prime \prime }\left(i,j\right)=\text{o}{\text{n}}_{h}^{\prime }\left(i,j\right)-\underset{m,n\in N\left(i,j\right)}{\sum }w_{m,n}\times \text{o}{\text{n}}_{h}^{\prime }\left(m,n\right),$$

where weighting factor *w*_*m*,*n*_ is

(3) $$w_{m,n}=\frac{1}{\left(\sqrt{{\left(i-m\right)}^{2}+{\left(j-n\right)}^{2}}+\varepsilon \right)},$$

where *ε* is a constant.

Central lateral inhibition mechanism of off channel is similar to on channel.

**(3) Correlation of Opposite Polarity Channels**

Channel on and channel off of small target-detected neurons correspond to the two edges of small targets, respectively; by shifting the opposite polarity channel, the small target detection results can be obtained after the correlation of opposite polarity channels. According to the small target definition in SPIE [20], the two edges of the small target are separated by 1 or 2 pixels. For the targets of different types (light or dark targets), the channel polarity which is selected to shift is different. The small targets in multi-spectral remote sensing images often have the characteristics of high spectral intensity and low probability of emergence and can be approximated as the light target, namely, *I*(*i*, *j*) > *I*(*i* + Δ, *j* + Δ). Thus, the signal polarity in the horizontal direction from left to right is channel on (increased brightness) and channel off (decreased brightness). Then, the channel-correlation processing of the remote sensing image is

(4) $$\text{output}\left(i,j\right)=\text{of}{\text{f}}_{h}^{\prime \prime }\left(i,j\right)\times \text{shift}\left(\text{o}{\text{n}}_{h}^{\prime \prime }\left(i,j\right),2\Delta \right),$$

where shift(·) is the shifting function, Δ is the shifting amount, Δ > 0 represents shifting right, and Δ < 0 represents shifting left.

#### 4.3.2. Small Target Detected Neuron Simulation and Analysis

**Figure 5:**
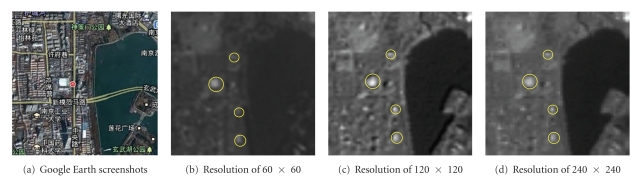
Experiment results of multispectral remote sensing image of Xuanwu district in Nanjing city.

**Figure 6:**
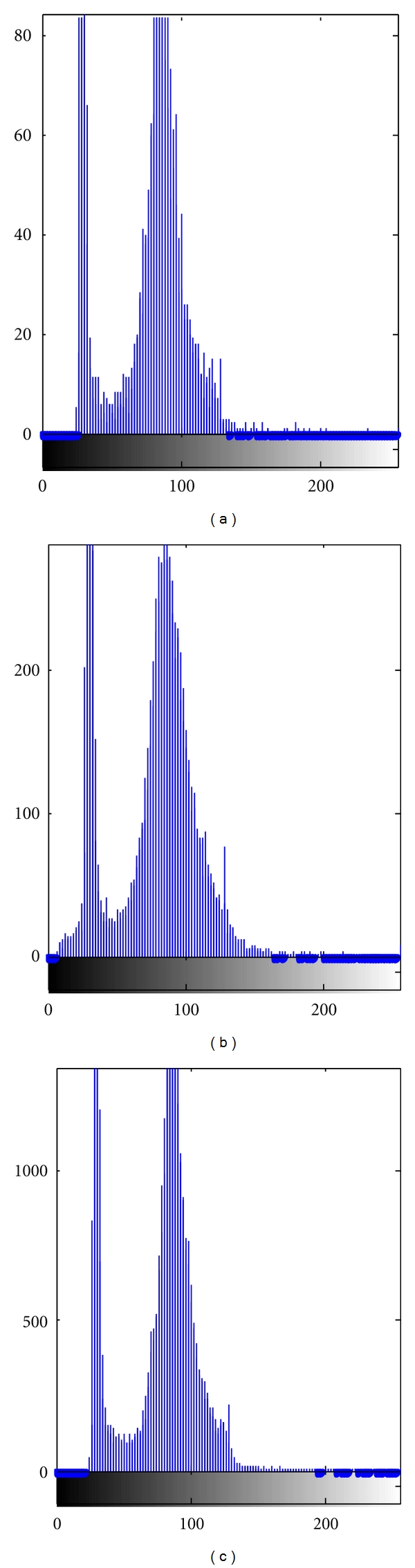
Background histogram under three different kinds of spatial resolution.

The local area with the size of 60 × 60 on the 3-band of the multispectral remote sensing image in Xuanwu District, Nanjing, obtained by Landsat satellite is used for the research. The area is the city's commercial district. A large number of buildings are concrete structures having poor absorption capacity and high reflection capacity, which are the light targets in remote sensing image.  Figure 5(a) is the corresponding region satellite images intercepted from the Google Earth as a reference for target location. Figures 5(b), 5(c), and 5(d) are remote sensing images with different spatial resolution, and the yellow circles mark the four preselected targets.  Figure 6 is the spectral distribution histogram in three types of spatial resolutions. There are two peaks in the spectral distribution of the image: the single peak of brightness has less energy in the lower brightness region, and the single peak appearing in the high brightness region is similar to the Gaussian distribution model but contains more energy.

**Figure 7:**
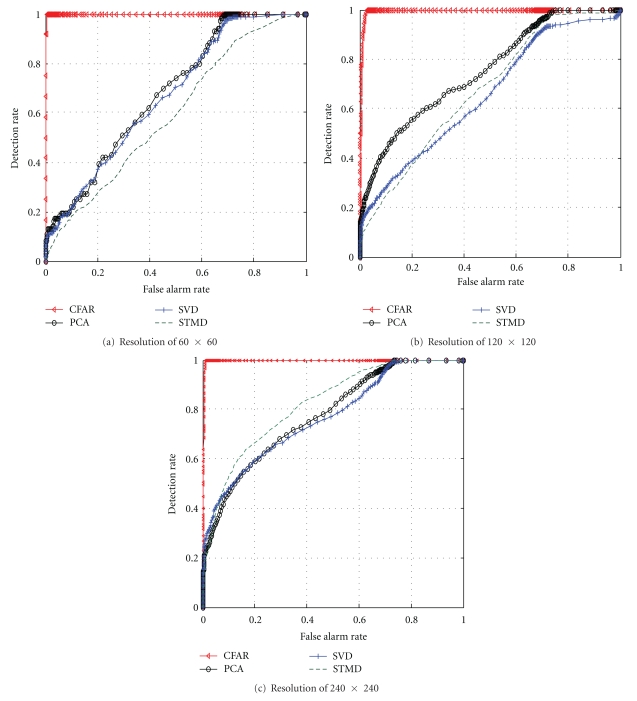
Comparison of false alarm rate of four detection algorithms under different spatial resolutions.

Figure 7 is the ROC curve under the conditions of four kinds of different spatial resolution detection algorithms. Green dashed line is the algorithm proposed by this paper, the red triangle line is CFAR algorithm, the black line ○ is PCA algorithm, and blue line + is SVD algorithm. Figures 7(a), 7(b), and 7(c) show comparison with the false alarm rates, respectively.

Spatial resolution CFAR algorithm always maintains a low level of false alarm rate, but SVD, PCA, and the proposed algorithms have almost similar false alarm rates. Combined with the spectral distribution histogram of the region, we can see that the background spectral distribution is similar to Gaussian distribution according to the theoretical basis of CFAR algorithm. So a lower false alarm rate of detection result can be obtained. As the spatial resolution increases, the false alarm rates of PCA, SVD, and the proposed algorithm decrease, where the false alarm rate of the proposed method declines fastest, although this algorithm shows that the false alarm rate is still higher than that of the CFAR algorithm. With the continuous improvement of spatial resolution images, background complexity increases, and the adaptive processing advantages will be fully reflected.

## 5. Conclusion

As the high precision and resolution remote sensing images in spatial and spectral observation scale result in complex background spectrum changes and target features diversity, the traditional large-scale-based target detection methods are difficult to transplant and apply. Current computer vision-based high-resolution remote sensing small target detection methods are either inhibiting complex background features from the background spectral analysis or describing and detecting targets by machine learning from the target features. Both aspects need to be improved in terms of the false alarm rate, real-time efficiency, robustness, and complexity of the algorithm.

Fly visual system has their unique advantages on small target detection in identification and tracking of natural scenes. Inspired by the information acquisition and processing mechanism of fly compound eye, this paper proposes small target detection fly-imitated visual information processing system pattern of high-resolution remote sensing images. Based on this model, the small target detector and fly-imitated visual perception algorithm are designed, providing a new strategy to further solve the difficulties existing in the automatic interpretation of high-resolution remote sensing image.
